# The Maverick Disease: Cystic Echinococcosis in Unusual Locations: A Ten Year Experience from an Endemic Region

**DOI:** 10.7759/cureus.5939

**Published:** 2019-10-18

**Authors:** Ayesha Butt, Javaid Khan

**Affiliations:** 1 Medical College, Aga Khan University, Karachi, PAK; 2 Internal Medicine: Pulmonology, Aga Khan University, Karachi, PAK

**Keywords:** hydatid disease, neglected tropical diseases, parasitic diseases, zoonotic diseases, echinococcus granulosus infection

## Abstract

Background

Cystic echinococcosis (CE) is a neglected tropical disease which affects more than 1 million people globally, causing a loss of 1-3 million disability-adjusted life years (DALYs) and a financial burden of US$ 3 billion annually.The two most commonly involved organs are the liver and the lungs with involvement in 75% and 5 -15% of cases respectively. The rest of the body can be involved in up to 10% of cases. In this study, we aim to explore the presentation, treatment and outcomes of CE in unusual locations.

Methods

Retrospective review of charts of 225 patients of CE admitted at Aga Khan Hospital, Karachi from 2007-2017 was done. Demographic information, date of admission, clinical presentation, laboratory and radiological findings, histopathology reports (where applicable), treatment course and outcomes were noted.

Results

CE occurred in the liver in 146 (64.9%) patients, in the lungs in 55 (24.4%) patients and in unusual locations in 24 (10.7%) patients. Primary involvement of unusual locations was seen in 22 (91.7%) cases. Amongst the 24 patients with disease in unusual locations, 13 (54.2%) were males and 11 (45.8%) were females and the median age of these patients was 43 years. Fever and dyspnea were the most common presenting complains, occurring in 5 (20.8%) patients each followed by epigastric abdominal pain and weight loss occurring in 3(12.5%) patients each. Spleen was the most common unusual location for CE with four cases (16.7%) of splenic involvement occurring, followed by cardiac, spinal and mediastinal involvement occurring in three (12.5%) patients each. Other unusual locations included the abdominal cavity, bones, breast, kidney, seminal vesicle, brain, adrenal glands and the inguinal region. The treatment courses employed were a) medical, consisting of oral albendazole use(400 mg twice daily), b) surgical c) combined (medical plus surgical) therapy. Combined surgical and medical therapy, was the most common modality employed, with it being given to 14 (58.3%) patients. Surgery only was performed in 5 (20.8%) patients while medical therapy only to 3 (12.5%) patients. Resolution of the disease was seen in 19 (79.2%) patients on follow up imaging. Recurrence occurred in 4 (16.7%) and mortality in 1 patient. Two patients (8.3%) were lost to follow up.

Conclusions

CE can be challenging to diagnose especially when it occurs in unusual locations. CE must be included in the differential diagnosis of a cystic lesion in any organ of the body, especially in endemic areas, to ensure timely diagnosis and treatment, to prevent morbidity and mortality associated with chronicity of the disease.

## Introduction

Echinococcosis is a zoonotic parasitic disease caused by the larvae of dog and fox tapeworms of *Echinococcus*. Cystic echinococcosis (CE), also known as hydatid disease or hydatidosis, and alveolar echinococcosis (AE) are the two major types of the disease, caused by* E. granulosus* and *E. multilocularis* respectively. CE is much more common than AE and is classified as a neglected tropical disease (NTD). Although present globally, CE is highly endemic in the Mediterranean region, northern Africa, at the southern tip of South America, southern and eastern Europe, in Central Asia, western China and Siberia. More than 1 million people globally are afflicted by CE, causing a loss of 1-3 million disability-adjusted life years (DALYs) and a financial burden of US$ 3 billion annually. Dogs serve as the definitive hosts and sheep, cattle, goats, and pigs as the intermediate hosts in the life cycle of *E. granulosus*. Dogs consume organs of other animals infested with hydatid cysts, which develop into adult tapeworms in dogs. Tapeworm eggs are then shed in feces of dogs and humans acquire the disease by ingestion of soil, water or food polluted with dog feces. Cystic echinococcosis was included by The World Health Organization (WHO) in a subdivision of selected NTDs to be tackled within its 2008-2015 strategic plan to control NTDs and WHO is now working towards establishing evidence based, impactful CE control strategies by 2020 [1].

Although CE can lead to formation of hydatid cysts in any part of the human body, the liver, followed by lungs are two organs classically involved in the disease [2]. However, in up to 10% of all cases, CE may occur in a kaleidoscope of unusual locations such as the spleen, heart, peritoneum, urinary tract, adrenal glands, ovary, brain, spinal cord, bone and soft tissues [3]. It is pertinent to study CE in unusual locations because CE diagnosis which is often missed even in the characteristic locations, becomes even more challenging when CE occurs in unusual locations. We aim to explore the unusual locations in which CE occurred in patients presenting to our center over a ten year period (2007-2017) and the presentation, treatment and outcomes of the disease in these patients.

## Materials and methods

The hospital records of 225 consecutive patients with a diagnosis of CE admitted at Aga Khan University Hospital, Karachi, from 2007-2017 were reviewed. 

A diagnosis of CE had been made in the patients based on clinical history, laboratory and radiological findings and response to treatment, and confirmed by histopathological evaluation of samples in cases were surgical intervention was done. A diagnosis of CE depends on clinical manifestations, imaging and serology. The clinical criteria for diagnosis of CE requires presence of one of the following: slowly growing or static cystic mass(es) visible on imaging, anaphylaxis as a result of rupture or leakage of cysts or incidental finding of a cyst by imaging techniques. The diagnostic criteria comprises of: characteristic organ lesion(s) detected by radiological techniques, high-sensitivity serological tests confirmed by a separate high specificity serological test, histopathology or parasitology findings corroborating CE and pathognomonic gross appearance of cyst(s) in surgical specimens.

Demographic information, date of admission, clinical presentation, laboratory and radiological findings, histopathology reports (where applicable), treatment course and outcomes were noted in a questionnaire. For quantitative variable analysis IBM SPSS version 23 was used. Simple frequencies and proportions were calculated. The data was collected after attaining exemption from the Ethical Review Committee (ERC) of the Aga Khan University Hospital (ERC number 2019-1921-5281).

## Results

A total of 225 patients with cystic echinococcosis were admitted in Aga Khan University Hospital over a ten year period. The patients included 126 males (56%) and 99 females (44%).There were 146 (64.9%) cases of hepatic hydatid cysts, 55 (24.4%) cases of pulmonary hydatid cysts and 24 (10.7%) cases with unusual localizations of hydatid cysts.

Among the 24 patients with disease in unusual locations, 13 (54.2%) were males and 11 (45.8%) were females. The median age of these patients was 43 years (SD 13.9), with the greatest proportion of patients belonging to the 41-60 years age group (n=11, 45.8%) (Table 1).

**Table 1:** Descriptive characteristics of patients with CE in unusual locations: demographics, diagnostic modalities, treatment and outcomes (n=24)

CHARACTERISTIC	FREQUENCY	PERCENTAGE (%)
AGE GROUPS, YEARS		
0-20	2	8.3
21-40	9	37.5
41-60	11	45.8
> 60	2	8.3
SEX		
Male	13	54.2
Female	11	45.8
EOSINOPHILIA		
Absent	22	91.7
Present	2	8.3
ELISA FOR ANTI-ECHINOCOCCOSIS ANTIBODIES		
Positive	6	25
Negative	3	12.5
Not done	15	62.5
RADIOGRAPHIC INVESTIGATIONS AVAILABLE		
Ultrasound	6	25
Computerised Tomography(CT) Scan	8	33.3
X-Ray	1	4.2
Magnetic Resonance Imaging(MRI)	2	8.3
TREATMENT		
Combined(Medical therapy+Surgery)	14	58.3
Surgery only	5	20.8
Medical therapy only	3	12.5
OCCURRENCE OF COMPLICATIONS	4	16.7
RECURRENCE	4	16.7
LOST TO FOLLOW UP	2	8.3
MORTALITY	1	4.2

Primary involvement of unusual locations was seen in 22 (91.7%) cases. Only two patients had concomitant involvement of the liver in addition to CE in unusual locations. The unusual locations in which CE was seen is enlisted in Table 2. Fever and dyspnea were the most common presenting complains, occurring in 5 (20.8%) patients each followed by epigastric abdominal pain and weight loss occurring in 3 (12.5%) patients each.

**Table 2:** Unusual Locations of Cystic Echinococcus in the body (n=24)

Location	Number of Cases	Percentage of total
Spleen	4	16.7
Heart	3	12.5
Spine	3	12.5
Mediastinum	3	12.5
Abdominal cavity	2	8.3
Kidney	1	4.16
Kidney + Liver	1	4.16
Left suboccipital region	1	4.16
Left Tibia	1	4.16
Right paracardiac +bilateral adrenal glands+left inguinal region.	1	4.16
Right breast and chest wall	1	4.16
Right Femur	1	4.16
Right Hip Joint (Right proximal femur, acetabulum and greater sciatic notch)	1	4.16
Seminal vesicle + Liver	1	4.16

Septic shock occurred in one patient. Two of the patients with splenic cysts presented with epigastric pain. The patient with a left suboccipital cyst presented with facial palsy, vomiting, vertigo and dizziness. Loss of appetite occurred in one patient with abdominal cysts while the other presented with melena. Dyspnea was the most common complain in patients with mediastinal cysts. Patients with cysts in the chest wall and the left tibia both presented with localized swelling. Moreover, whereas one patient with right hip involvement experienced localized pain, another one with proximal femur involvement presented with a discharging sinus. All patients with spinal involvement had lower limb weakness while one of them had urinary incontinence as well.

Upon workup, eosinophilia was present in only 2 (8.3%) patients. The Echinococcus antibody Immunoglobulin G (IgG) assay which is an enzyme-linked immunosorbent assay (ELISA) based qualitative detection of IgG antibodies was used in 9 (37.5%) patients. ELISA for anti-echinococcus antibodies was more than 1:16 in 6 (25%) patients and less than 1:16 in 3 (12.5%) patients (Table 1). A CT scan was performed in 8 (33.3%) patients while ultrasound was done in 6 (25%). Imaging revealed a solitary cyst in 4 (16.7%) patients.

The treatment courses employed were a) medical, consisting of oral albendazole use(400 mg twice daily), b) surgical c) combined (medical plus surgical) therapy. Combined surgical and medical therapy, was the most common modality employed, with it being given to 14 (58.3%) patients. Surgery only was performed in 5 (20.8%) patients while medical therapy only to 3 (12.5%) patients.

Complications during treatment course occurred in 4 (16.7%) patients. These complications included: Left ear discharge in the patient who underwent craniotomy for left suboccipital hydatid cysts, copious and clear wound discharge for which a lumbar drain was inserted in a patient with spinal CE and postoperative fever in two other patients.

Resolution of the disease was seen in 19 (79.2%) patients on follow up imaging. Recurrence occurred in 4 (16.7%) and mortality in 1 patient. Two patients (8.3%) were lost to follow up.

## Discussion

CE is grave public health issue afflicting populations spread across many different parts of the world and leading to significant morbidity and financial costs associated with treatment and the chronic nature of the disease [2]. The two most commonly involved organs are the liver and the lungs with involvement in 75% and 5 -15% of cases respectively [3].The rest of the body can be involved in up to 10% of cases [4]. According to our findings as well, the liver emerged as the most common organ affected, comprising 64.9% of the total cases followed by pulmonary hydatid cysts in 24.4% of the cases whereas 10.7% cases had unusual localization.

Our results showed a greater percentage of male patients whereas previous studies from Pakistan have reported the male to female ratio in CE patients as 1:1.2 and 1:2.14 [5]. A systematic review of literature has also previously delineated CE to be more prevalent in females (Prevalence Proportion Ratio: 1.35 [95% Bayesian Credible Interval: 1.16-1.53]) [6]. In contrast, in our study, the ratio of males to females was 1: 0.79.

CE can cause cysts in any location in the body. A review delineated the most common unusual locations to be the central nervous system, musculoskeletal system, heart and kidney, whereas some less common locations included the spleen, pancreas, appendix, thyroid, salivary gland, adrenal gland, breast, and ovary [7].

CE afflicts the spleen in 0.5% to 11.3% of all CE cases and is considered to be the third most prevalent site of CE [2]. Hydatid cysts in the spleen can arise as a consequence of systemic spread due to rupture of a primary cyst at another location. Nonetheless, primary hydatid cysts can arise in the spleen, albeit very rarely [8-10]. In our study, cysts in the spleen were seen in 16.7% of cases, making it the most frequently involved unusual site of CE. All of these patients had isolated splenic involvement with hepatic and pulmonary involvement being excluded by imaging.

Osseous involvement by CE occurs in 0.5% to 2.5% of all CE patients and spinal involvement occurs in almost half of the cases [2]. The bone becomes primarily involved as a corollary of the settlement of blood-borne scolexes in bone. The proliferation of the cyst usually follows the bone canals and they may mimic locally malignant lesions on imaging. Hence, a final diagnosis can only be made by pathological assessment [2, 11]. According to our results, six (25%) patients had osseous CE, including patients with cysts in hip joint, femur, tibia and spine and half the cases involved the spine.

CE involves the central nervous system in 1% to 3% of patients and manifests as a space occupying lesion in the brain usually [2]. Intracranial hydatidosis is more frequently seen than spinal hydatidosis [12, 13]. The lesions are usually solitary and most often occur in the area supplied by the middle cerebral artery, particularly the parietal lobe. Brain hydatid cysts occur more frequently in children and are supratentorial [3, 14]. Amongst our patients, only one had a cerebral cyst, which was located in the subocciptal area.

CE in the urogenital system are rare. Renal CE is seen in only 1% to 4% of all CE cases [2]. Renal cysts may present a diagnostic challenge as they are difficult to differentiate from mass lesions and/or acquired cystic diseases of the kidney. In addition, these cysts are usually solitary, located at the upper pole or cortex and may reach sizes of up to 10 cm before producing any symptoms [2]. The route of infection of CE to the urinary tract is believed to be through the retroperitoneal lymphatics. A palpable mass, flank pain,malaise, hematuria, fever are common presentations while lower back pain is the most common symptom of renal CE [15]. The two patients in our study presented with lower back pain and decreased appetite respectively. Hydatid cysts in the seminal vesicles are extremely rare with scarce reports in literature [16-18]. Amongst our patients, only one had seminal vesicle involvement.

Hydatid cysts can develop in the heart in 0.2-2% of patients with CE [3, 14]. They may be a ramification of rupture of a lung hydatid cyst or may occur via spread by blood. Transthoracic echocardiography, computerized tomography (CT), and magnetic resonance imaging (MRI) can be employed for diagnosis. The most commonly affected cardiac areas are the left ventricle (50-60% of cases), interventricular septum (10-20%), the right ventricle (5-15%), pericardium (10-15%), and the right or left atrium (5-8%) [3].

 CE in the adrenal gland is also uncommon, and is usually a corollary of disseminated disease, as was the case in the one patient with adrenal involvement in our study, who had paracardiac, inguinal and adrenal cysts. The differential diagnosis of adrenal hydatid lesions includes endothelial cysts, pseudocysts, lymphangiomatous and angiomatous cysts and cystic degeneration of adrenal neoplasm [19].

Breast is an exceedingly rare location for CE to develop and accounts for only 0.27% of all cases [20, 21]. Mammography shows vague findings and the cyst usually appears as a circumscribed mass with thin peripheral calcification, similar to a simple cyst. On ultrasound, the lesion is usually indistinguishable from simple or complicated cysts [20, 21]. On MRI, it appears as a well defined cystic lesion with capsular enhancement [20, 21]. In our study, we encountered only one case of hydatid cyst in the breast and chest wall.

CE can be managed through four main modalities: medical therapy, percutaneous methods e.g. Puncture, Aspiration, Injection, Re-aspiration (PAIR) procedure , surgery and “wait and watch” [22]. Surgery is the treatment of choice for complicated, large or infected cysts, cysts at high risk of rupture and cysts in important organ locations [23]. Among our patients a combined management approach, consisting of surgery and albendazole was used in the majority (58.3%) of patients.

Anti-parasitic medical therapy with benzimidazoles (BMZ), mainly albendazole, is effective for treating uncomplicated cysts, cysts in the liver or lungs in inoperable patients, cases with numerous cysts in two or more organs or peritoneal cysts. Moreover, pulmonary and hepatic CE1 and CE3a cysts less than 5cm in diameter respond favorably to BMZ alone [23]. BMZ is also important in preventing recurrence following surgery or PAIR. However, BMZs alone should not be used against large cysts (>10 cm) and should be avoided in early pregnancy or against cysts likely to rupture [23]. A Watch and Wait approach is reserved for uncomplicated CE4 and CE5 cysts [23].

Serology has only a minor, confirmatory role due to a high rate of false negative results [23]. This is the reason why ELISA for anti-echinococcosis antibodies was done in only 37.5% of our patients. In the remaining patients, diagnosis was based on clinical presentation, radiological findings and histopathological evaluation of resected samples where applicable. Results of radiological investigation were accessible for only 17 out of 24 of our patients. The remaining patients had imaging reports from other center with them at time of presentation. The details of these imaging reports could not be accessed in our retrospective review conducted at our center, however, mention of imaging reports from other centers was found in charts of these 7 patients.

## Conclusions

Cystic echinococcus can be challenging to diagnose especially when it occurs in unusual locations. Hence it is important to be cognizant that CE may be seen in any organ in the human body and must be included in the differential diagnosis of a cystic lesion in any organ especially in endemic regions to ensure timely diagnosis and treatment.
